# Associations of Retinal Curvature With Choroidal Thickness and OCTA-Derived Choroidal Flow-Density Metric in High Myopia: A Two-Center OCTA Study of Interocular Asymmetry

**DOI:** 10.1167/tvst.15.5.26

**Published:** 2026-05-28

**Authors:** Ze-xu Wang, Bin Wei, Rui Li

**Affiliations:** 1 Medical College of Wuhan University of Science and Technology, Wuhan, People's Republic of China; 2 Department of Ophthalmology, Xiaogan Hospital Affiliated to Wuhan University of Science and Technology, the Central Hospital of Xiaogan, Xiaogan, People's Republic of China; 3 The First Affiliated Hospital, Jiangxi Medical College, Nanchang University, Nanchang, Jiangxi, People's Republic of China

**Keywords:** high myopia, retinal curvature, choroidal thickness, optical coherence tomography angiography

## Abstract

**Purpose:**

The purpose of this study was to investigate the relationship between RC and choroidal structural and perfusion-density metrics in high myopia, and to determine whether interocular asymmetry is more closely associated with structural differences or optical coherence tomography angiography (OCTA)-derived perfusion differences.

**Methods:**

In this two-center cross-sectional study, participants with and without high myopia underwent OCTA imaging. RC (10^−2^ mm^−2^), choroidal thickness (CT, µm), and OCTA-derived choroidal flow-density metric (CF, %) were quantified within six concentric macular rings centered on the fovea. Eye-level associations between the RC and choroidal metrics were evaluated using generalized estimating equation models adjusting for age, sex, axial length (AL), and imaging center. Interocular (within-subject) asymmetry was primarily assessed using participant-level interocular difference analyses, with linear regression models evaluating associations between ΔRC and corresponding differences in ΔCT and ΔCF after adjustment for interocular AL difference and imaging center. Additional Spearman correlation analyses were performed as nonparametric robustness checks. Sensitivity analyses and measurement repeatability assessments were performed to evaluate robustness.

**Results:**

At the eye level, a steeper RC was significantly associated with reduced choroidal thickness across multiple macular rings, with the strongest association observed in the parafoveal region (ring 3), followed by the central macula (ring 1), and weaker associations toward the peripheral retina (ring 6). These associations remained significant after multivariable adjustment and correction for multiple comparisons. Associations between RC and CF density were weaker and more region-dependent: significant RC main effects were observed in selected rings (notably ring 3 and ring 6) in the eye-level analysis, whereas these associations were not consistently supported in the interocular analysis after multivariable adjustment and correction for multiple comparisons. Interocular analyses demonstrated that ΔRC was strongly and consistently associated with ΔCT, again with the largest effect observed in ring 3. These findings were robust across sensitivity analyses for CT, whereas the corresponding results for CF density were less consistent across analytical approaches. RC and CT measurements exhibited excellent repeatability, with intraclass correlation coefficients (ICCs) exceeding 0.98.

**Conclusions:**

RC was consistently associated with CT at both the eye level and in interocular comparisons, whereas associations with the OCTA-derived CF density metric were weaker, region-dependent, and not consistently supported in interocular analyses. These findings suggest that interocular asymmetry in high myopia is primarily associated with structural differences, with less consistent involvement of the OCTA-derived flow-density metric assessed in this study.

**Translational Relevance:**

OCTA-based RC assessment may provide a clinically accessible framework for regional evaluation of posterior pole structure in high myopia; however, further validation in longitudinal and multi-platform studies is required.

## Introduction

High myopia is a leading cause of irreversible visual impairment worldwide and is frequently accompanied by profound structural alterations of the posterior pole.^1^^–^^4^ Beyond excessive axial elongation, highly myopic eyes often exhibit complex and regionally heterogeneous structural alterations of the retina and choroid, particularly in the macular area. Increasing evidence from prior imaging and observational studies suggests that localized posterior pole structural changes may be associated with disease severity and visual prognosis, beyond global ocular size alone.^2^^,^^5^

Among these structural alterations, deformation of the posterior pole has been proposed as an important structural feature of myopic eyes. Traditional metrics such as axial length (AL) provide a global measure of ocular elongation but fail to capture localized geometric changes of the posterior segment.^5^^–^^7^ In contrast, retinal curvature (RC) offers a region-specific descriptor of posterior pole shape, may capture localized geometric variation not reflected by AL alone, and has been hypothesized to relate to localized biomechanical stress and tissue change.^8^^–^^11^ Prior studies using magnetic resonance imaging (MRI)-based eye models have demonstrated asymmetric posterior pole geometry and interocular differences in highly myopic individuals, underscoring the importance of posterior pole shape in myopia-related pathology.^9^^–^^13^

The choroid, a highly vascularized tissue critical for retinal metabolism and thermoregulation, undergoes marked thinning in high myopia and has been implicated in the pathogenesis of myopic maculopathy.^14^^,^^15^ Choroidal thickness (CT) has been shown to vary regionally across the macula and to differ substantially between fellow eyes in asymmetric myopia. However, whether these choroidal changes are closely linked to posterior pole geometry remains incompletely understood. In particular, it remains unclear whether localized curvature alterations are associated with changes in choroidal structure, optical coherence tomography angiography (OCTA)-derived perfusion-density metrics, or both.^14^^–^^17^

Recent advances in OCTA have enabled noninvasive, high-resolution assessment of both CT and OCTA-derived choroidal flow (CF)-density metric in vivo.^16^^–^^19^ Unlike MRI, OCTA is widely available in clinical settings, allows repeated measurements, and provides layer-specific structural and perfusion information. These advantages make OCTA a promising modality for studying posterior pole structural alterations and their functional correlates in myopia.^9^^–^^11^^,^^20^ Importantly, OCTA-based approaches may allow curvature-related geometric features to be quantified at a regional level, facilitating investigation of structure–structure and structure–perfusion relationships within the same imaging framework.^10^^,^^20^^,^^21^

Another unresolved question concerns interocular asymmetry in high myopia. Fellow eyes within the same individual often differ in AL, posterior pole shape, and choroidal characteristics. Interocular comparisons provide a useful within-subject design that reduces confounding by shared systemic factors such as age, sex, and genetic background, although local ocular differences between fellow eyes may still contribute to interocular asymmetry. Nevertheless, few studies have systematically examined whether interocular differences in RC are coupled with corresponding differences in choroidal structure or perfusion across macular regions.^22^^–^^25^

Therefore, the purpose of this study was to investigate the relationship between RC and choroidal characteristics in high myopia using OCTA-derived measurements. Specifically, we aimed to (1) assess the association among RC and CT and CF density across concentric macular rings, (2) evaluate whether these associations differ between eye-level and interocular analyses, and (3) determine whether curvature-related interocular asymmetry is more closely linked to structural differences or to the OCTA-derived CF density metric assessed in this study. By integrating regional curvature metrics with choroidal structural and perfusion-density data, this study seeks to clarify geometric–structural associations of the posterior pole in high myopia and to establish a clinically accessible framework for assessment.

## Methods

### Study Population

This dual-center observational study was conducted at Xiaogan Central Hospital and the First Affiliated Hospital of Nanchang University. The two participating centers used identical recruitment criteria, ophthalmic examination workflows, imaging acquisition procedures, measurement workflows, and examination timing protocols. The study adhered to the principles of the Declaration of Helsinki, and the study protocol was approved by the Institutional Review Board/Ethics Committee (approval number: KY–2025102201). Written informed consent was obtained from all participants prior to enrollment.

Inclusion criteria comprised patients age 20 to 40 years, bilateral intraocular pressure (IOP) ≤21 millimeters of mercury (mm Hg), refractive error of −6.00 diopter (D) < SE ≤ −0.50 D for non-high myopia participants, or SE ≤ −6.00 D or AL ≥26.0 mm for high myopia participants, consistent with commonly adopted AL-based definitions used in epidemiologic and imaging studies of high myopia and aligned with International Myopia Institute recommendations,^5^^,^^26^ best-corrected visual acuity (BCVA) ≥0.8, image quality score ≥8, and voluntary informed consent to participate. Exclusion criteria included any ocular disease other than myopia (e.g. cataract, glaucoma, diabetic retinopathy, or uveitis), prior retinal laser treatment, or ocular surgery. Participants with systemic chronic diseases (e.g. diabetes, hypertension, and hyperthyroidism) were excluded. Eyes with media opacity, motion artifacts, or segmentation artifacts that impaired reliable imaging were excluded.

### Ophthalmic Examination and Imaging

All participants underwent a series of ophthalmic examinations, including medical history collection, IOP measurement, cycloplegic refraction, slit-lamp biomicroscopy, dilated fundus examination, measurement of ocular biometric parameters, and swept-source OCTA (SS-OCTA) imaging. Biometric parameters, such as AL, anterior chamber depth (ACD), vitreous thickness (VT), and mean keratometry (Km), were obtained using an ophthalmic biometer. SS-OCT and OCTA scans were acquired using a 400 kilohertz (kHz) SS-OCTA system (BM-400K BMizar, Tupai Medical Technology, Beijing, China) with a 1060-nm vertical-cavity surface-emitting laser (VCSEL), scanning speed of 400,000 A-scans/s, axial resolution of 3.8 µm, and lateral resolution of 10 µm. A single ultra-widefield 24 × 20 mm scan centered on the fovea (1536 A-scans × 1280 B-scan positions; A-scan depth 6.0 mm [2560 pixels]) was obtained for each eye, providing a field of view of up to 120 degrees; 2 consecutive B-scans were acquired at each location to support flow detection and improve signal stability. Before curvature quantification, OCT volumes underwent motion-artifact reduction and manual review. AL-based magnification correction was applied to each eye prior to regional quantification. The automatically segmented Bruch's membrane was visually inspected by an experienced grader in each eye, and manual correction was performed when necessary. Eyes with image artifacts or segmentation errors that could not be reliably corrected and that affected posterior pole surface reconstruction were excluded from analysis. A fitted three-dimensional retinal surface model was generated based on the reconstructed Bruch's membrane, and pointwise curvature radius values (r) were computed across the full 24 × 20 mm region (1536 × 1280 points); the magnitude of r reflects local surface steepness (larger |r| indicates flatter curvature, whereas smaller |r| indicates steeper curvature).

### Retinal Curvature Measurement

**Figure 1.**
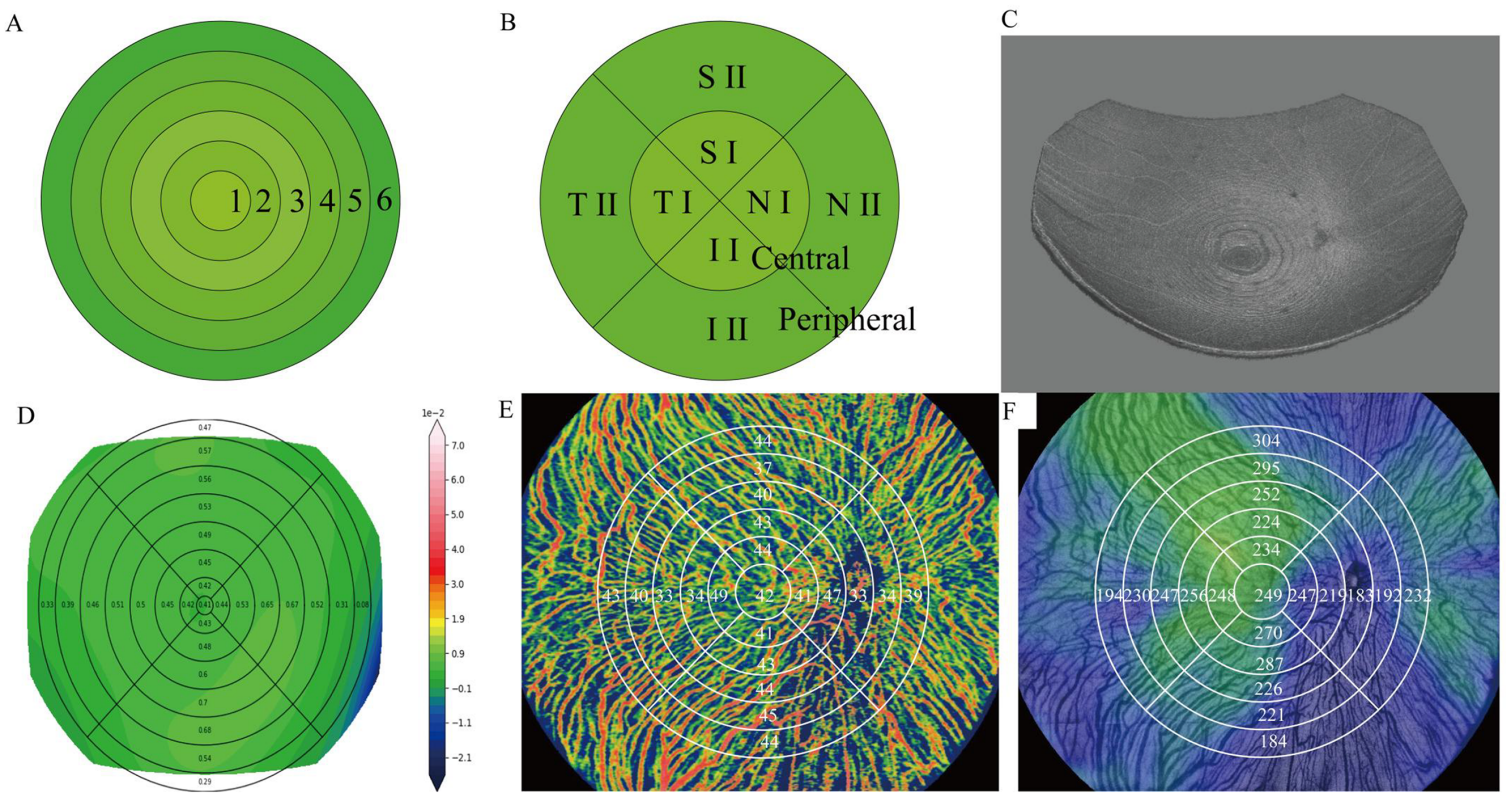
**Retinal curvature** **-** **based regional framework and multimodal choroidal metrics.** (**A**) Schematic illustration of concentric ring-based segmentation used for retinal curvature (RC), choroidal thickness (CT), and choroidal flow-density metric (CF) analyses. The posterior pole was divided into six concentric rings centered on the fovea (ring 1–ring 6), from the central to peripheral retina. (**B**) Sector-based subdivision of the posterior pole into central and peripheral regions, further partitioned into superior, inferior, nasal, and temporal quadrants for regional analyses. (**C**) Three-dimensional reconstruction of the posterior retinal surface derived from optical coherence tomography (OCT), illustrating global retinal shape. (**D**) Representative RC map showing spatial variation in retinal curvature across concentric rings and quadrants. (**E**) En face CF-density metric map overlaid with the concentric ring grid, illustrating regional differences in choroidal perfusion. (**F**) En face CT map with concentric ring segmentation, demonstrating spatial heterogeneity of choroidal thickness across the posterior pole. For visualization purposes, ring boundaries and labels in panels **E** and **F** are overlaid on OCTA-derived maps and may be partially obscured by background signal intensity. CF, OCTA-derived choroidal flow-density metric; CT, choroidal thickness; OCT, optical coherence tomography; RC, retinal curvature.

RC data within the Early Treatment Diabetic Retinopathy Study (ETDRS) grid centered on the macula were generated using the device’s built-in algorithms (Fig. 1). Because of potential folding or incomplete capture in peripheral regions, the analysis was limited to a 15-mm diameter circular area centered on the macula. The central 1-mm zone was designated as region 1, whereas data from the remaining regions were reorganized into concentric annuli with outer diameters of 3 mm, 6 mm, 9 mm, 12 mm, and 15 mm, sequentially labeled regions 2 to 6 (see Fig. 1A). This structure was designed to analyze retinal morphological changes from the macula to the periphery. The macular curvature was defined as the area-weighted average curvature of regions 1, 2, and 3, whereas the peripheral curvature was determined from regions 4, 5, and 6 (see Fig. 1B). Furthermore, quadrants based on nasal, inferior, temporal, and superior directions were used to calculate RCs in the macular and peripheral areas, labeled NI, II, TI, and SI (center) and NII, III, TII, and SII (peripheral), respectively (see Fig. 1B). The same regional framework was used for CT and CF density. In this study, CF represents a device-derived OCTA flow-density metric, calculated as flow-positive vascular area divided by the selected region area and expressed as a percentage (%), rather than as an absolute physical measure of blood flow (see Fig. 1).

**Figure 2.**
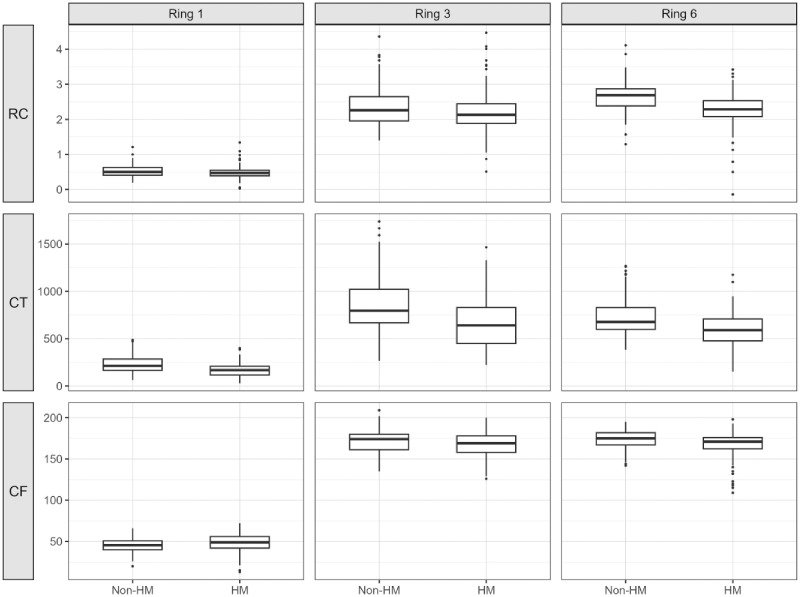
**Comparison of retinal curvature, choroidal thickness, and choroidal flow-density metric between highly myopic and non** **-** **highly myopic eyes.** Box-and-whisker plots showing distributions of retinal curvature (RC), choroidal thickness (CT), and choroidal flow-density metric (CF) across concentric macular rings (ring 1, ring 3, and ring 6) in high myopia (HM) and non-high myopia (non-HM) eyes. A total of 288 eyes from 144 participants were included in the eye-level analyses. *Boxes* indicate the interquartile range (IQR), *horizontal lines* represent medians, whiskers extend to 1.5 × IQR, and individual points denote individual eyes. Group differences were formally evaluated using generalized estimating equation (GEE) models accounting for inter-eye correlation and adjusted for age and sex, with additional adjustment for axial length in sensitivity analyses. Corresponding adjusted *P* values are reported in Supplementary Table S5. GEE, generalized estimating equation; HM, high myopia; IQR, interquartile range.

### Repeatability and Reliability

All SS-OCTA scans were performed by an experienced technician following standardized procedures. Each subject underwent examination in a dark room between 2:00 PM and 5:00 PM after resting quietly for 15 minutes prior to the examination. Short-term repeatability of RC and CT at ring 1 and ring 6 were evaluated in a subset of eyes undergoing repeated OCTA imaging across 2 consecutive days. Each eye was scanned three times on the first day and once on the second day. Intraclass correlation coefficient (ICC; 2-way random-effects model and absolute agreement) were calculated to evaluate repeatability. Detailed results are provided in the Supplementary Material. Because RC is derived from a global Bruch's membrane surface reconstruction rather than from independent ring-based primary acquisitions, repeatability is primarily determined by the stability of the underlying segmentation and surface-fitting process.

### Statistical Analysis

All statistical analyses were performed using R software (version 4.5.1; R Foundation for Statistical Computing, Vienna, Austria). Continuous variables approximating a normal distribution are presented as mean ± standard deviation, whereas skewed variables are summarized as median (interquartile range), based on distributional assessment. Eye-level associations between RC and choroidal metrics were evaluated using generalized estimating equation (GEE) models to account for correlation between fellow eyes while preserving eye-level observations. Given the two-center study design, the imaging center was included as a covariate in all multivariable models. Models were additionally adjusted for age, sex, and AL where applicable. To assess within-subject asymmetry, interocular analyses were performed using participant-level interocular difference variables, defined as the value in the longer AL eye minus that in the shorter AL eye. Associations between ΔRC and corresponding differences in ΔCT or ΔCF were evaluated using participant-level linear regression models with adjustment for interocular AL difference (ΔAL) and imaging center. Because interocular differences were not normally distributed, additional Spearman correlation analyses were performed as nonparametric robustness checks. These analyses were intended to confirm that the observed associations were not dependent on distributional assumptions of the regression models. Because each participant contributed a single paired observation, mixed-effects models would not materially alter the inference structure of these participant-level analyses. The robustness of interocular associations was further assessed using trimming, winsorization, and alternative correlation methods where appropriate. Measurement repeatability was evaluated using ICC based on a two-way random-effects model. When multiple ring-wise comparisons were performed within the same analysis, false discovery rate (FDR) correction was applied using the Benjamini–Hochberg procedure. All statistical tests were two-sided, and a *P* value < 0.05 was considered statistically significant unless otherwise specified. A total of 288 eyes from 144 participants across 2 imaging centers were included in the final analysis, with 144 paired participants available for interocular analyses.

## Results

### Study Population and Baseline Characteristics

A total of 288 eyes from 144 participants were included in the analysis. At the eye level, 146 eyes were classified as highly myopic (HM) and 142 as non-highly myopic (non-HM). For participant-level baseline comparisons, 77 participants were classified as HM (defined as having at least one HM eye) and 67 as non-HM. Baseline demographic and ocular characteristics are summarized in Table 1, and group-wise distributions of retinal curvature, choroidal thickness, and choroidal flow-density metric are shown in Figure 2. Eyes with HM, defined as an AL ≥26.0 mm or spherical equivalent ≤−6.0 D, exhibited longer AL and thinner CT than non-HM eyes. Center-stratified descriptive comparisons of choroidal thickness by high myopia status are shown in Supplementary Fig. S1. The 26.0-mm AL threshold was chosen because it is commonly used in AL-based epidemiologic and imaging studies and aligns with International Myopia Institute classification frameworks.

**Table 1.** Baseline Characteristics of the Study Participants

Characteristic	Overall	Non-HM	HM	*P* Value
Age	32.00 [25.00 to 37.00]	33.00 [23.00 to 37.00]	32.00 [27.00 to 38.00]	0.300
Sex				0.150
Female	91 (63%)	47 (70%)	44 (57%)	
Male	53 (37%)	20 (30%)	33 (43%)	
Center				0.074
Center 1	91 (63%)	48 (72%)	43 (56%)	
Center 2	53 (37%)	19 (28%)	34 (44%)	
AL	25.60 [24.76 to 26.59]	24.66 [24.01 to 25.21]	26.55 [25.86 to 27.48]	**<0.001**
SE	−6.00 [−7.75 to −3.00]	−3.00 [−4.00 to −2.00]	−7.50 [−9.50 to −6.50]	**<0.001**
IOP	15.0 [13.1 to 16.7]	14.9 [13.0 to 16.7]	15.0 [13.1 to 16.8]	0.600
ACD	3.72 [3.53 to 3.90]	3.70 [3.48 to 3.89]	3.74 [3.54 to 3.90]	0.400
VT	17.97 [17.12 to 18.91]	17.12 [16.53 to 17.76]	18.89 [18.24 to 19.59]	**<0.001**
K1	42.99 [42.19 to 43.80]	42.94 [42.19 to 43.77]	42.99 [42.19 to 44.06]	0.800
K2	43.83 [42.91 to 44.50]	43.83 [42.78 to 44.29]	43.83 [42.94 to 44.94]	0.400
Km	43.42 [42.51 to 44.24]	43.42 [42.49 to 43.96]	43.36 [42.57 to 44.40]	0.600
WTW	11.79 [11.51 to 12.01]	11.78 [11.51 to 12.03]	11.80 [11.50 to 11.97]	0.900

ACD, anterior chamber depth; AL, axial length; IOP, intraocular pressure; K1, flat keratometry; K2, steep keratometry; Km, mean keratometry; SE, spherical equivalent; VT, vitreous thickness; WTW, white-to-white distance.

Values are presented as median [interquartile range] for continuous variables and number (percentage) for categorical variables. High myopia (HM) was defined as an axial length ≥26.0 mm or a spherical equivalent ≤ −6.00 diopters. *P* values were calculated using the Mann–Whitney *U* test for continuous variables and the chi-square test for categorical variables.

The *P* values in bold represent statistical significance.

### Associations Between Retinal Curvature and Choroidal Metrics at the Eye Level

**Figure 3.**
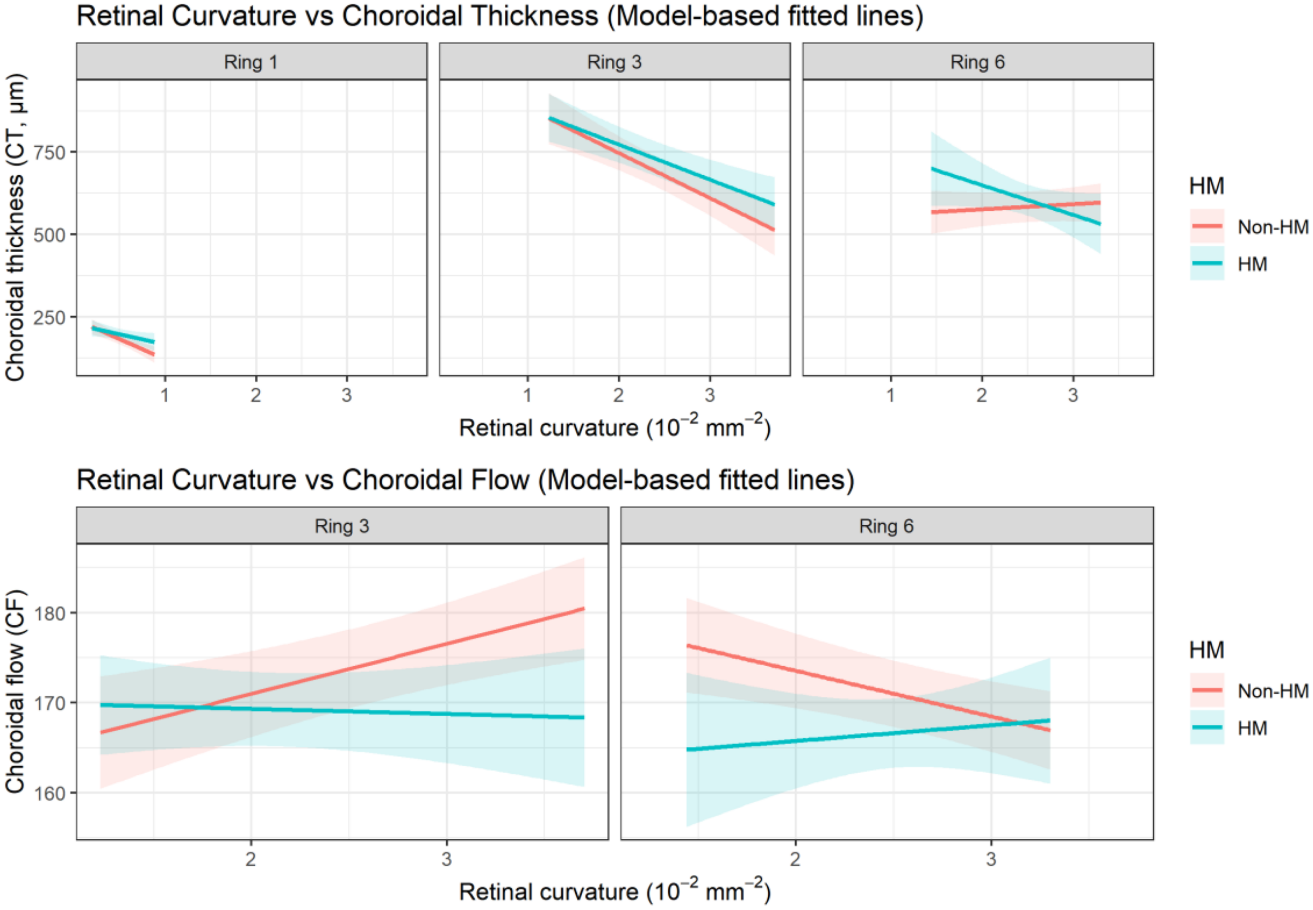
**Associations** **between** **posterior** **retinal** **curvature and** **choroidal** **thickness and** **choroidal** **flow-density** **metric** **across** **concentric** **rings.** Model-based fitted lines depicting the associations between posterior retinal curvature (RC) and choroidal thickness (CT) (*top row*) and choroidal flow-density metric (CF) (*bottom row*) across concentric macular rings. A total of 288 eyes from 144 participants were included in the eye-level analyses. Curves represent predictions from eye-level generalized estimating equation (GEE) models accounting for inter-eye correlation (participant ID as the clustering variable; exchangeable working correlation). Separate fitted lines are shown for high myopia (HM) and non-high myopia (non-HM), with shaded bands indicating 95% confidence intervals. Models were adjusted for age, sex, axial length, and study center. CT is shown for ring 1, ring 3, and ring 6. Associations between RC and the CF-density metric were weaker and region-dependent, with significant RC main effects observed in the reference group in selected rings (ring 3 and ring 6), whereas these associations were not consistently supported in interocular analyses. RC is expressed in 10 to 2 mm^−2^; CT is expressed in µm; CF is expressed as a percentage (%). CI, confidence interval; OCTA, optical coherence tomography angiography.

Eye-level associations between RC and choroidal metrics were evaluated using GEE models accounting for inter-eye correlation. Across concentric macular rings, steeper RC was consistently associated with reduced CT. This association was strongest in ring 3, followed by ring 1, and was weaker toward the peripheral retina (ring 6; Table 2; Fig. 3). In Table 2, the RC main effect (β1) represents the association between RC and the outcome in the reference group (non-HM), whereas the RC × HM interaction term (β3) indicates whether this association differs in HM eyes. These relationships remained statistically significant after adjustment for age, sex, AL, and study center, as well as after correction for multiple comparisons using the FDR procedure. Standardized effect size estimates for the primary eye-level associations are provided in Supplementary Table S9. In contrast, associations between RC and CF density were weaker and more region-dependent than those observed for CT. In the eye-level analysis, significant RC main effects for CF density were observed in selected rings after multivariable adjustment and FDR correction (see Table 2; Fig. 3). However, these associations were not consistently supported in the interocular analysis after multivariable adjustment and correction for multiple comparisons. Overall, RC–CF associations were confined to selected regions and were less consistent than the corresponding RC–CT associations. No significant interaction between RC and HM status was observed for CT or CF across rings after FDR correction, indicating that the RC–choroid associations were broadly consistent between highly myopic and non-highly myopic eyes (see Table 2). Center-stratified fitted associations and formal RC × center interaction analyses are shown in Supplementary Fig. S2. Formal RC × center interaction results are provided in Supplementary Table S8.

**Table 2.** Associations of Posterior Retinal Curvature With Choroidal Thickness and the OCTA-Derived Choroidal Flow-Density Metric Across Concentric Rings

Outcome Measure	Model Term	Ring 1 β [95% CI]	Ring 3 β [95% CI]	Ring 6 β [95% CI]	Ring 1 *P* Value	Ring 3 *P* Value	Ring 6 *P* Value	Ring 1 *q* Value (FDR)	Ring 3 *q* Value (FDR)	Ring 6 *q* Value (FDR)
CT	RC main effect (β1)	−122.82 [−174.24 to −71.40]	−136.62 [−186.76 to −86.47]	15.68 [−28.08 to 59.43]	<0.001	<0.001	0.482	<0.001	<0.001	0.482
CT	RC × HM interaction (β3)	61.45 [−12.58 to 135.49]	30.14 [−34.94 to 95.21]	−105.83 [−201.77 to −9.89]	0.104	0.364	0.031	0.156	0.364	0.092
CF	RC main effect (β1)	7.24 [−2.19 to 16.68]	5.56 [2.28 to 8.83]	−5.07 [−8.33 to −1.81]	0.132	<0.001	0.002	0.132	0.003	0.003
CF	RC × HM interaction (β3)	−1.28 [−14.05 to 11.49]	−6.12 [−11.35 to −0.89]	6.81 [−0.39 to 14.02]	0.844	0.022	0.064	0.844	0.065	0.096

CF, OCTA-derived choroidal flow-density metric; CI, confidence interval; CT, choroidal thickness; FDR, false discovery rate; GEE, generalized estimating equation; HM, high myopia; RC, retinal curvature.

Multivariable associations of posterior retinal curvature (RC, 10−2 mm^−2^) with choroidal thickness (CT, µm) and OCTA-derived choroidal flow-density metric (CF, %) across ring 1, ring 3, and ring 6. Estimates are from eye-level generalized estimating equation (GEE) models with an exchangeable working correlation structure and participant ID as the clustering variable to account for inter-eye correlation. Models included RC (main effect, β1), high myopia status (HM), and the interaction term RC × HM (β3), adjusted for age, sex, axial length, and study center. β1 represents the association between RC and the outcome in the reference group (non-HM). β3 represents effect modification by HM (the additional RC effect in HM relative to non-HM). For each ring, results are reported as β (95% CI), nominal *P* value, and false discovery rate-adjusted *q* value. The *q* values were calculated using the Benjamini–Hochberg false discovery rate (FDR) procedure across the ring-wise tests within each outcome/term family. Associations between retinal curvature and choroidal flow density were weaker and region-dependent, with significant RC main effects were observed in the reference group in selected rings (ring 3 and ring 6), whereas these associations were not consistently supported in interocular analyses.

### Interocular Asymmetry Analyses

**Figure 4.**
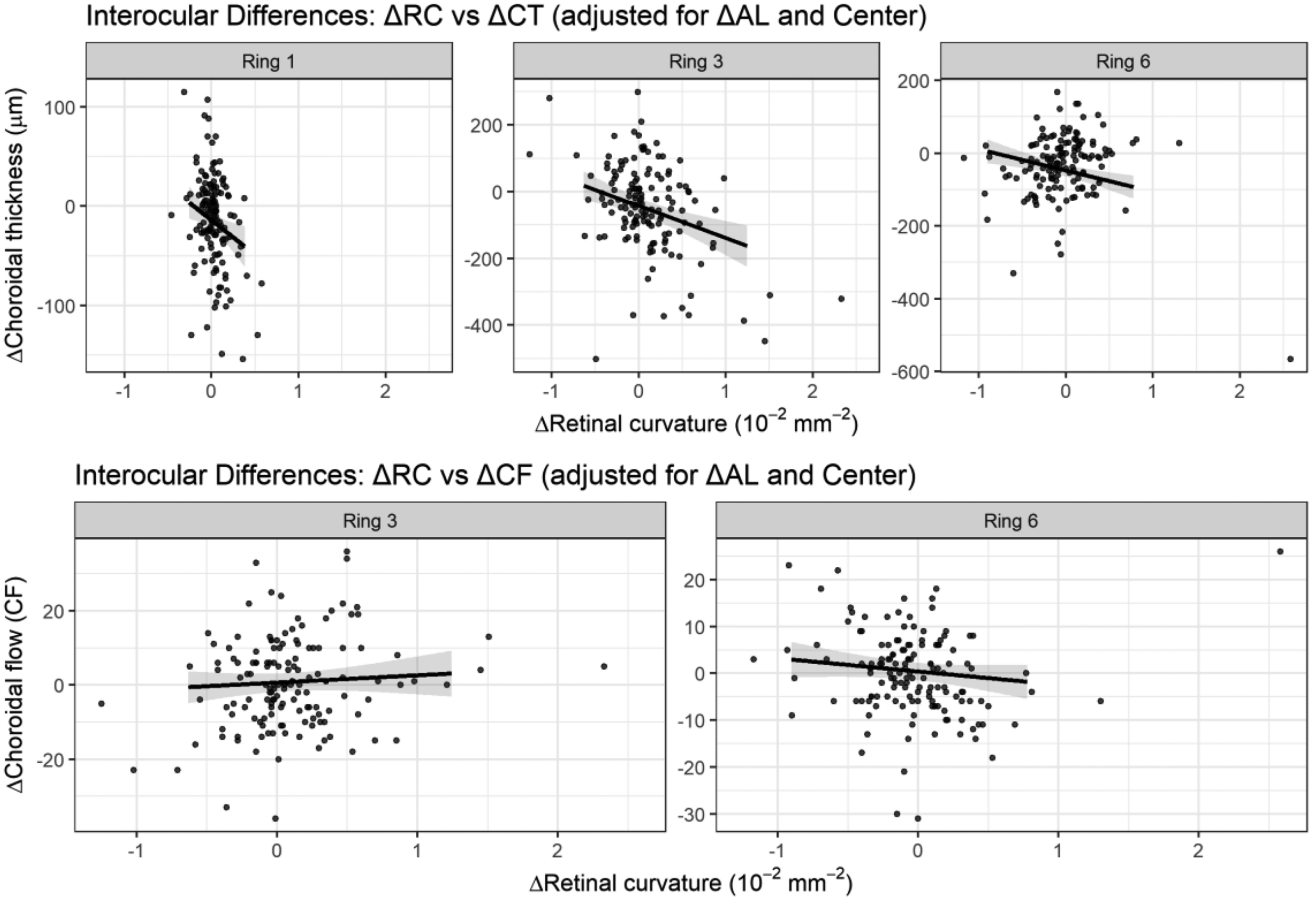
**Interocular** **differences in** **retinal** **curvature** **are** **associated** **with** **interocular** **differences in** **choroidal** **thickness but** **not** **consistently** **with** **choroidal** **flow-density** **metric.** Interocular difference (Δ) analysis illustrating within-participant associations between retinal curvature asymmetry and choroidal asymmetry. Each point represents one participant. A total of 144 paired participants were included in the interocular difference analysis. With interocular differences computed as Δ = (long eye − short eye), where the long eye was defined by longer axial length. The *top row* shows ΔRC versus ΔCT for ring 1, ring 3, and ring 6; the *bottom row* shows ΔRC versus ΔCF for ring 3 and ring 6. *Solid lines* indicate adjusted linear fits and shaded areas represent 95% confidence intervals. Models were adjusted for ΔAL and study center. Quantitative regression results are reported in Table 3. ΔRC is expressed in 10⁻² mm⁻²; ΔCT in µm; ΔCF in instrument-derived units. AL, axial length.

To assess within-subject asymmetry, interocular differences (Δ = longer AL eye minus shorter AL eye) were calculated for RC, CT, and CF. Interocular differences in RC (ΔRC) were significantly associated with corresponding differences in CT (ΔCT). The long eye was defined as the eye with the longer AL within each participant. Eyes with greater RC asymmetry exhibited greater CT asymmetry, with the strongest association observed in ring 3, followed by ring 1, and a weaker but still significant association in ring 6 (Table 3; Fig. 4). These associations remained significant after adjustment for interocular AL difference (ΔAL) and study center, and after FDR correction. In contrast, ΔRC was not significantly associated with interocular differences in CF (ΔCF) in rings 3 or 6 after multivariable adjustment and FDR correction (see Table 3; Fig. 4). Complete ring-wise interocular analyses are provided in Supplementary Table S2.

**Table 3.** Interocular Associations Between Retinal Curvature Differences and Choroidal Thickness and Flow-Density Metric

Outcome	Region (Ring)	Exposure	β (95% CI)	*P* Value	*q* Value (FDR)
ΔCT	Ring 1	ΔRC (α1)	−69.33 (−119.90 to −18.76)	**0.008**	**0.008**
ΔCT	Ring 3	ΔRC (α1)	−96.68 (−145.83 to −47.53)	**<0.001**	**<0.001**
ΔCT	Ring 6	ΔRC (α1)	−58.44 (−91.59 to −25.28)	**<0.001**	**0.001**
ΔCF	Ring 3	ΔRC (α1)	1.96 (−2.977 to 6.90)	0.438	0.438
ΔCF	Ring 6	ΔRC (α1)	−2.82 (−6.64 to 1.01)	0.151	0.302

ΔRC, interocular difference in retinal curvature; ΔCT, interocular difference in choroidal thickness; ΔCF, interocular difference in OCTA-derived choroidal flow-density metric; ΔAL, interocular difference in axial length; FDR, false discovery rate; OCTA, optical coherence tomography angiography.

Interocular difference (Δ) models evaluating whether within-participant differences in retinal curvature are associated with within-participant differences in choroidal thickness and choroidal flow density. For each participant, the “long eye” was defined as the eye with longer axial length (tie-breaker: right eye). Interocular differences were computed as Δ = (long eye − short eye) for retinal curvature (ΔRC), choroidal thickness (ΔCT), and choroidal flow density (ΔCF). Linear models were fitted at the participant level and adjusted for interocular axial length difference (ΔAL) and study center. Results are reported as β (95% CI), two-sided *P* value, and false discovery rate–adjusted *q* value. Retinal curvature is expressed in 10−2 mm−2; choroidal thickness is expressed in µm. Choroidal flow density (CF) is reported as a device-derived flow-density metric as exported from the OCTA device.

The *P* and *q* values in bold represent statistical significance.

### Quadrant-Based Exploratory Analyses

Exploratory quadrant-based analyses were performed to evaluate potential directional heterogeneity of interocular asymmetry. Interocular differences in CT demonstrated heterogeneous quadrant-specific associations, with more prominent asymmetry observed in inferior and superior-related sectors, whereas nasal and temporal sectors showed less consistent patterns. Associations involving CF were weaker and spatially inconsistent across quadrants. These findings are presented in Supplementary Table S3.

### Sensitivity and Robustness Analyses

The robustness of the main findings was confirmed in multiple sensitivity analyses. Analyses using summed central, peripheral, and total indices are provided in Supplementary Table S4. Nonparametric Spearman correlation analyses yielded consistent results with the primary models (Supplementary Table S6). Trimming and winsorizing extreme observations did not materially alter the direction or significance of the interocular RC–CT associations, indicating that the observed relationships were not driven by outliers (Supplementary Table S7, Supplementary Fig. S3). Additional models substituting AL with spherical equivalent produced similar estimates (Supplementary Table S10). A quartile-based visualization of the ring 3 retinal curvature associations with CT and CF is provided in Supplementary Fig. S4.

### Measurement Repeatability

Repeatability measurements were excellent, with ICCs exceeding 0.98 across the evaluated rings (ring 1 and ring 6) and centers. No evidence of systematic differences was observed across repeated scans (F = 0.005–0.026, all *P* > 0.99; Supplementary Table S1).

## Discussion

In this study, RC was closely associated with CT at both the eye level and in interocular comparisons, with the strongest associations observed in the parafoveal region. By contrast, associations between RC and the OCTA-derived CF density metric were weaker, region-dependent, and less consistent than those observed for choroidal thickness.^17^ Although selected eye-level models showed significant RC main effects for CF density, these findings were not consistently supported in interocular analyses. Together, these results suggest that interocular asymmetry in HM may be primarily associated with structural differences, with less consistent involvement of the OCTA-derived flow-density metric assessed here.

The present findings are consistent with the view that RC provides a measure of localized geometric variation of the posterior pole that is closely associated with choroidal structural differences. Unlike AL, which reflects global ocular elongation, curvature provides region-specific information on posterior pole shape and may be hypothesized to relate to localized biomechanical stress.^3^^,^^26^^–^^29^ The strongest associations observed in the parafoveal region (ring 3) are particularly notable. This region represents a biomechanical transition zone between the foveal center and the peripheral retina; however, any suggestion that this area is especially vulnerable during myopic eye growth remains speculative and hypothesis-generating, as temporal or developmental processes cannot be inferred from the current cross-sectional design. Furthermore, these localized geometric variations were consistently associated with corresponding differences in CT, suggesting a structure–structure association pattern between posterior pole geometry and choroidal structural changes.^30^^–^^33^ Despite these robust structural associations, the corresponding findings for the OCTA-derived CF density metric were weaker and more heterogeneous. Significant RC main effects were observed in selected eye-level regions, but these findings were not consistently supported in the interocular analysis and were less consistent across complementary analyses than the RC–CT associations. This pattern suggests that curvature-related differences were more consistently reflected in CT than in the specific flow-density metric assessed here.^34^^,^^35^ The less consistent CF findings may also partly reflect limitations of OCTA-derived flow-density metrics, which represent a density-based index rather than a direct measure of absolute blood flow.

Previous investigations of posterior pole geometry and ocular shape asymmetry have largely relied on MRI-based reconstructions. Although an MRI provides valuable three-dimensional information, its high cost, limited accessibility, and impracticality for routine or repeated examinations have restricted its widespread use in both clinical and research settings. In contrast, the present study supports the feasibility of quantifying RC using OCTA-derived structural data. OCTA is widely available in ophthalmic clinics, noninvasive, and suitable for repeated measurements, making curvature-based assessment of posterior pole geometry feasible at scale. The excellent repeatability observed in the repeated OCTA acquisitions and the stability of the segmentation-based reconstruction process support the robustness of this approach. By enabling localized geometric assessment using a clinically accessible imaging modality, this method substantially lowers the barrier to posterior pole structural alterations in myopia. Future longitudinal studies are needed to determine whether this approach can be used to monitor change over time.

A major strength of this study is the use of interocular (within-subject) comparisons, which reduce confounding from shared systemic factors such as age, sex, and genetic background. However, local ocular differences between fellow eyes, including asymmetric posterior pole configuration, scleral properties, or subclinical structural abnormalities, may still contribute to interocular asymmetry. The consistency of these associations across multiple robustness checks, including nonparametric correlations, trimming, and winsorization procedures, further supports the reliability of the observed findings. Several limitations should also be acknowledged. First, the cross-sectional design precludes inference regarding the temporal sequence of RC changes and associated choroidal structural alterations. Second, our strict exclusion of ocular comorbidities was intended to reduce heterogeneity but may limit generalizability to the broader population of highly myopic eyes encountered in clinical practice, in whom subtle degenerative or tractional changes are common. Finally, the OCTA-derived CF density metric analyzed here represents a density-based perfusion index expressed as a percentage rather than an absolute measure of blood flow. OCTA-based perfusion metrics may also be influenced by projection or decorrelation artifacts, limited sensitivity to subtle vascular alterations, inter-device non-equivalence, and segmentation-dependent bias, particularly in highly myopic eyes. In addition, repeatability was evaluated within the present device-specific acquisition and reconstruction workflow; therefore, direct extrapolation to other platforms or analytic implementations should be made cautiously.

In summary, RC was consistently associated with CT, particularly in the parafoveal region, whereas associations with the OCTA-derived CF density metric were weaker, region-dependent, and not consistently supported in the interocular analysis. Importantly, the ability to quantify posterior pole curvature using OCT-based imaging may provide a practical framework for studying myopia-related structural alterations in future clinical and research settings.

## Supplementary Material

Supplement 1

Supplement 2

Supplement 3

Supplement 4

Supplement 5

Supplement 6

Supplement 7

Supplement 8

Supplement 9

Supplement 10

Supplement 11

Supplement 12

Supplement 13

Supplement 14
