# Supplementary material for: Associations of Retinal Curvature With Choroidal Thickness and OCTA-Derived Choroidal Flow-Density Metric in High Myopia: A Two-Center OCTA Study of Interocular Asymmetry
Source: Transl Vis Sci Technol. 2026 May 28;15(5):26. doi: 10.1167/tvst.15.5.26 (PMC13225303; doi:10.1167/tvst.15.5.26)
Supplement: Supplement 1 [file tvst-15-5-26_s001.docx]

**Supplementary Figure S1. Center-Stratified Descriptive Comparison of Choroidal Thickness by High Myopia Status**


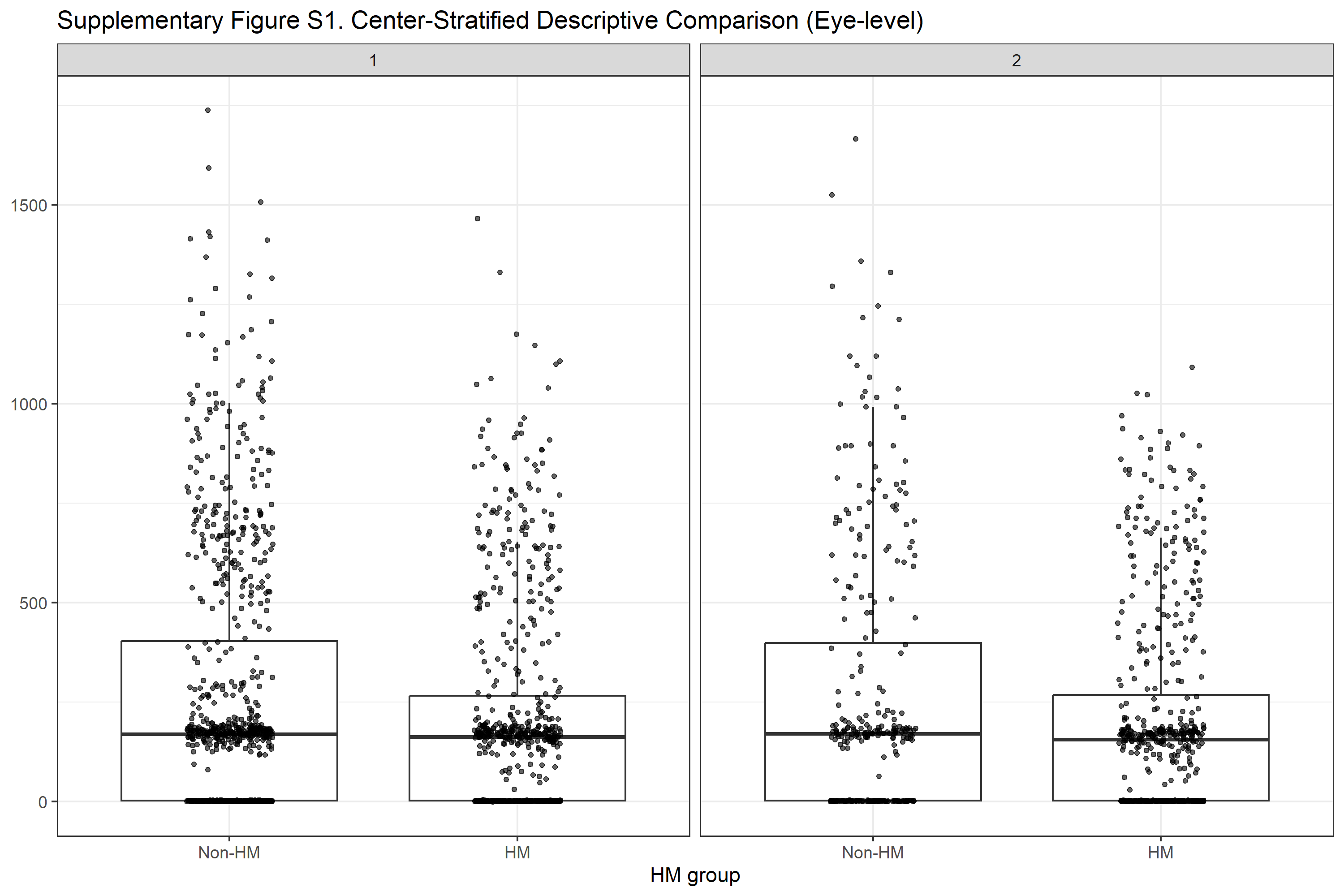


Center-stratified descriptive comparison of the eye-level distribution of choroidal thickness (CT) between non–high myopia (non-HM) and high myopia (HM) groups. Panels show data from **Center 1** and **Center 2** separately. Boxplots indicate the median (center line), interquartile range (box), and 1.5×IQR whiskers; points represent individual eyes (jittered for visibility). This figure is intended to assess whether the direction of the HM vs non-HM difference is visually consistent across study centers. N = 288 eyes from 144 participants (Center 1: 182 eyes; Center 2: 106 eyes).

## Abbreviations: HM = high myopia; non-HM = non–high myopia; CT = choroidal thickness; IQR = interquartile range.
