# Supplementary material for: Associations of Retinal Curvature With Choroidal Thickness and OCTA-Derived Choroidal Flow-Density Metric in High Myopia: A Two-Center OCTA Study of Interocular Asymmetry
Source: Transl Vis Sci Technol. 2026 May 28;15(5):26. doi: 10.1167/tvst.15.5.26 (PMC13225303; doi:10.1167/tvst.15.5.26)
Supplement: Supplement 2 [file tvst-15-5-26_s002.docx]

**Supplementary Figure S2. Interaction Between Retinal Curvature and Study Center for Choroidal Thickness and Choroidal Flow-Density Metric**


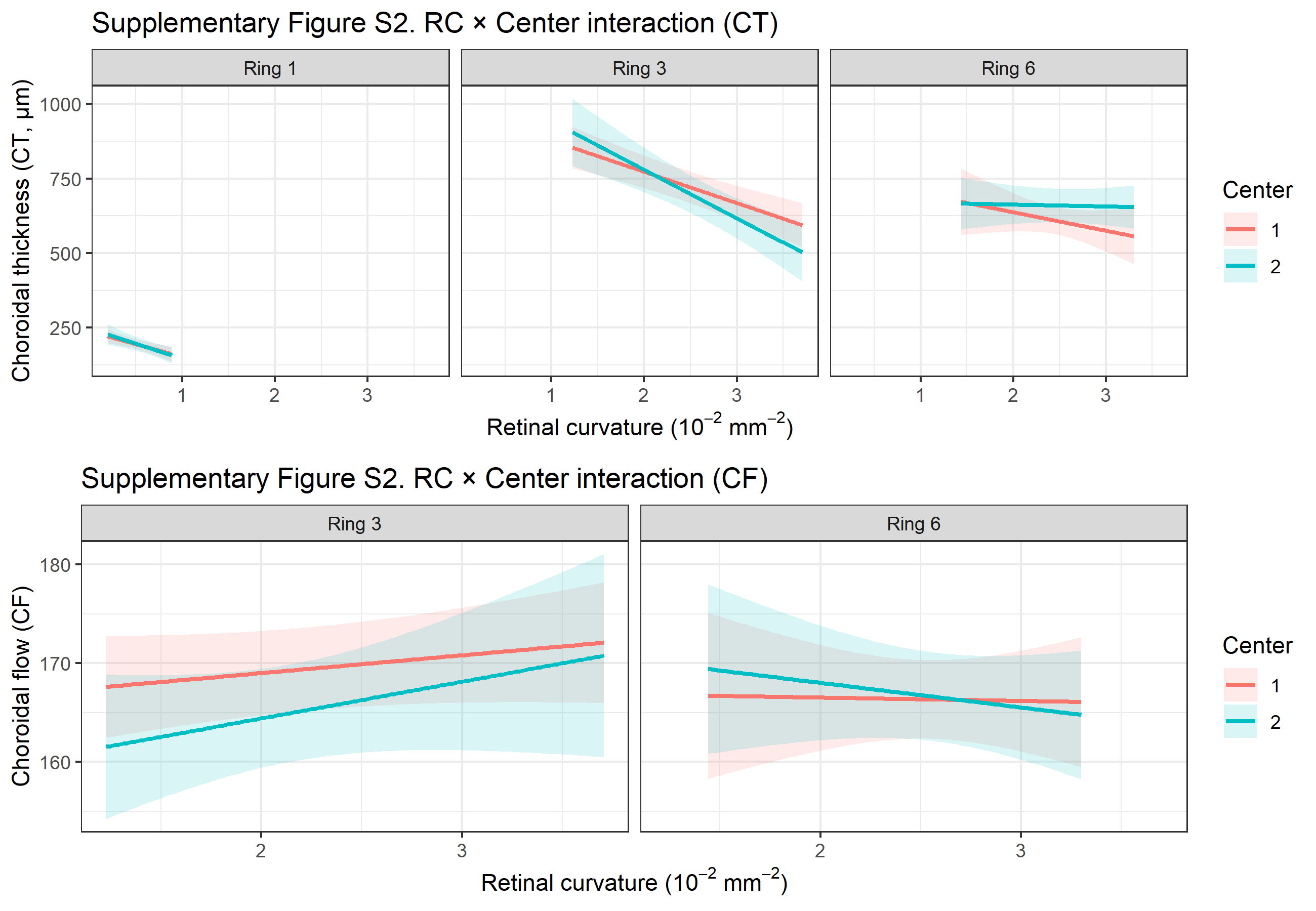


Model-based fitted relationships between retinal curvature (RC) and (Top) choroidal thickness (CT) and (Bottom) choroidal flow-density metric (CF), stratified by study center.Lines represent adjusted marginal predictions derived from generalized estimating equation (GEE) models including RC, center, and their interaction term, with adjustment for age, sex, axial length, and eye-level clustering. Shaded areas indicate 95% confidence intervals.No statistically significant RC × center interaction was observed for CT or CF across Rings 1, 3, or 6 (all P for interaction > 0.05), indicating that the association between retinal curvature and choroidal metrics was consistent across centers.

**Abbreviations:** RC = retinal curvature; CT = choroidal thickness; CF = OCTA-derived choroidal flow-density metric.
