# Supplementary material for: Associations of Retinal Curvature With Choroidal Thickness and OCTA-Derived Choroidal Flow-Density Metric in High Myopia: A Two-Center OCTA Study of Interocular Asymmetry
Source: Transl Vis Sci Technol. 2026 May 28;15(5):26. doi: 10.1167/tvst.15.5.26 (PMC13225303; doi:10.1167/tvst.15.5.26)
Supplement: Supplement 3 [file tvst-15-5-26_s003.docx]

## ****Supplementary Figure S3. Robustness Check for Ring 6: Interocular ΔRC6 Versus ΔCT6****


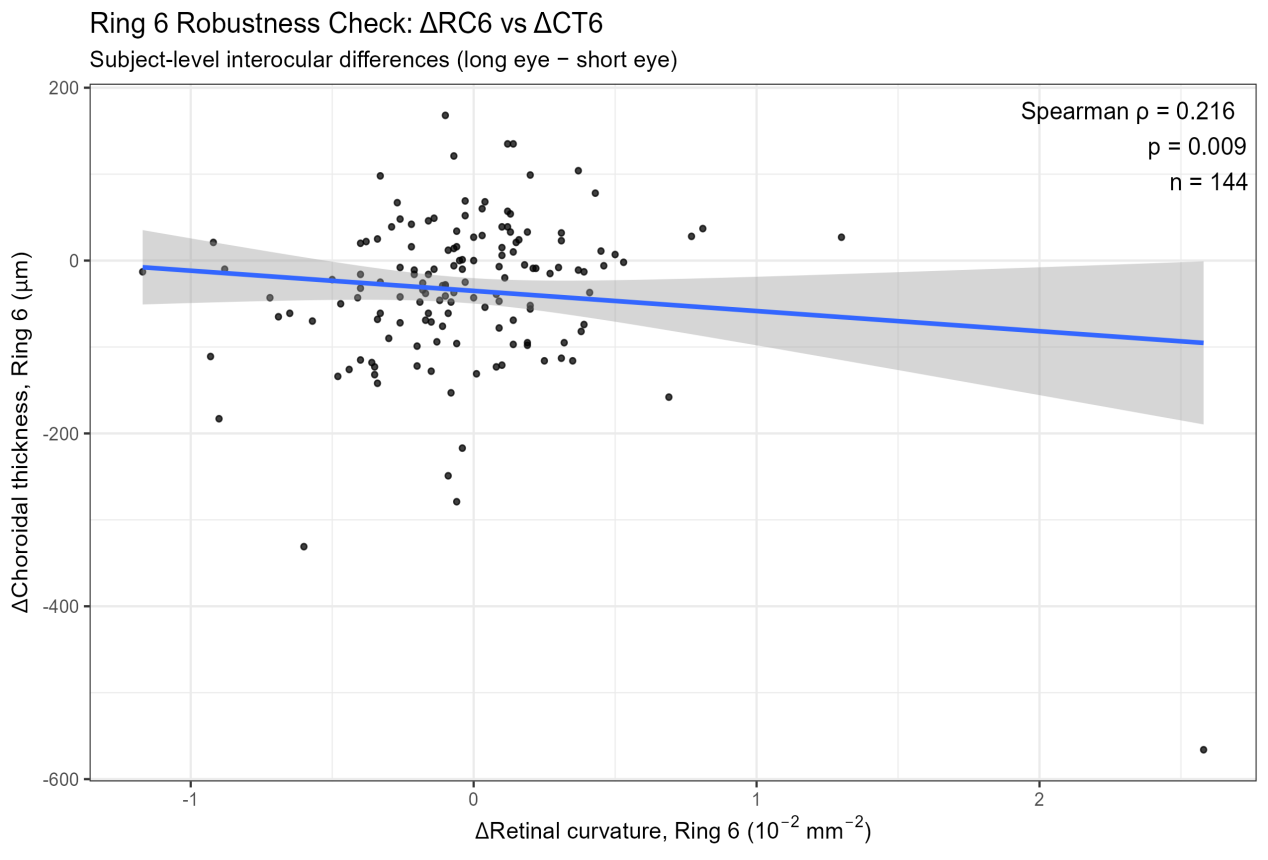


This figure is provided as a robustness check to evaluate whether the Ring 6 association is driven by extreme observations or non-linear patterns. Scatter plot showing the relationship between interocular differences in retinal curvature (ΔRC) and interocular differences in choroidal thickness (ΔCT) at Ring 6. Each point represents one participant, with interocular differences computed as **Δ = (long eye − short eye)**, where the long eye was defined as the eye with longer axial length. The solid line indicates the fitted linear trend with a 95% confidence band. Spearman rank correlation (ρ), P value, and sample size are displayed on the plot.

### Abbreviations: **ΔRC** = interocular difference in retinal curvature; **ΔCT** = interocular difference in choroidal thickness; **AL** = axial length.
