# Supplementary material for: Associations of Retinal Curvature With Choroidal Thickness and OCTA-Derived Choroidal Flow-Density Metric in High Myopia: A Two-Center OCTA Study of Interocular Asymmetry
Source: Transl Vis Sci Technol. 2026 May 28;15(5):26. doi: 10.1167/tvst.15.5.26 (PMC13225303; doi:10.1167/tvst.15.5.26)
Supplement: Supplement 4 [file tvst-15-5-26_s004.docx]

****Supplementary Figure S4. Dose–response of Ring 3 retinal curvature with choroidal thickness and choroidal flow-density metric by quartiles****


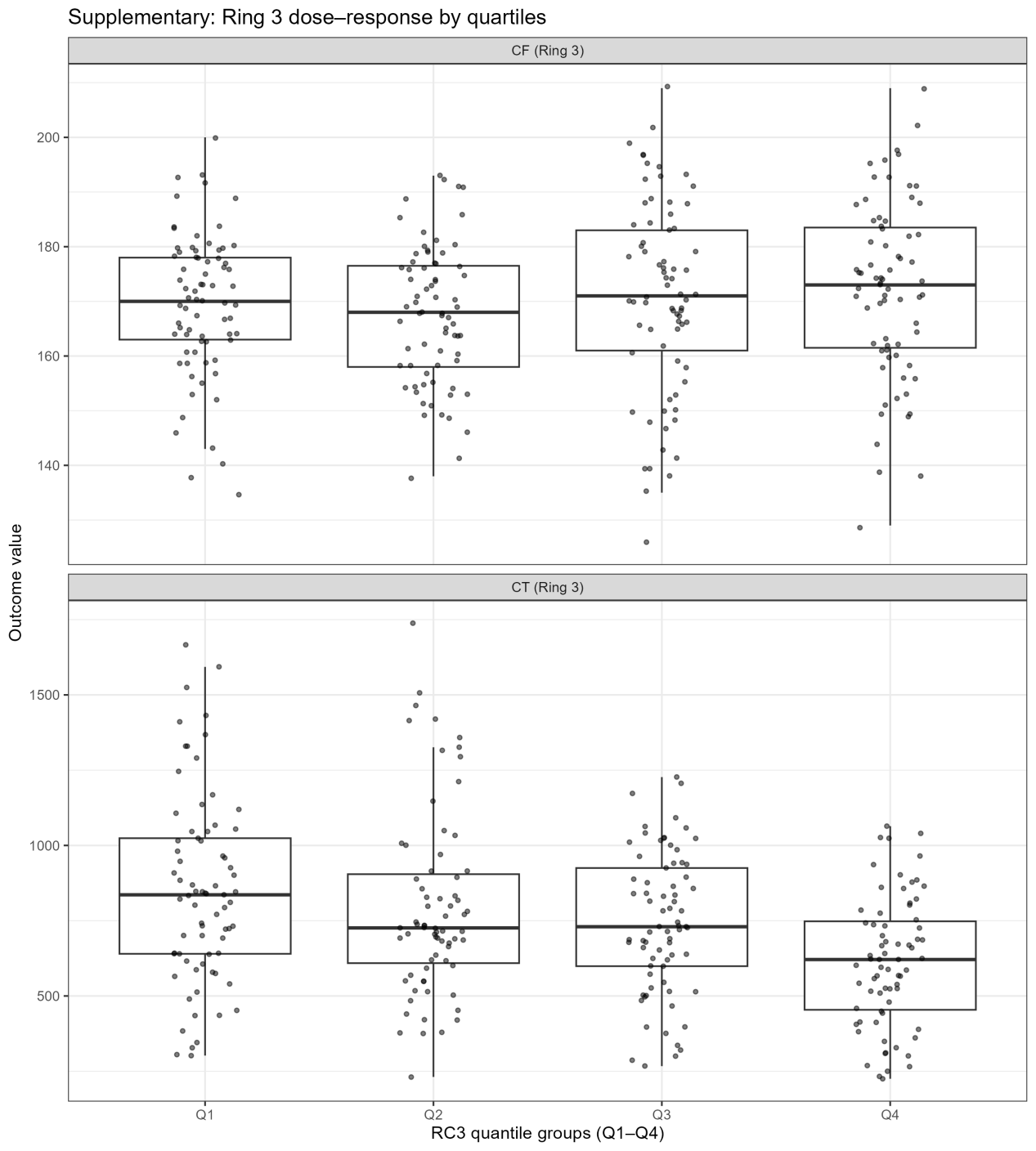


Boxplots with overlaid jittered points show distributions of choroidal flow-density metric **(CF, Ring 3)** and **choroidal thickness (CT, Ring 3)** across quartiles of **retinal curvature (RC, Ring 3)** (Q1–Q4). Quartile groups were defined based on the distribution of RC in Ring 3. **Q1 denotes the lowest RC quartile and Q4 the highest RC quartile.** This figure provides a nonparametric visualization of potential nonlinearity (threshold or monotonic trends) in the RC–choroid associations. Statistical inference for between-quartile differences and trend tests was obtained using eye-level generalized estimating equation models accounting for inter-eye correlation (participant ID clustered) and adjusted for age, sex, axial length, and study center.

**Abbreviations:** RC = retinal curvature; CT = choroidal thickness; CF = OCTA-derived choroidal flow-density metric; GEE = generalized estimating equation; AL = axial length; CI = confidence interval.
