# Supplementary material for: Associations of Retinal Curvature With Choroidal Thickness and OCTA-Derived Choroidal Flow-Density Metric in High Myopia: A Two-Center OCTA Study of Interocular Asymmetry
Source: Transl Vis Sci Technol. 2026 May 28;15(5):26. doi: 10.1167/tvst.15.5.26 (PMC13225303; doi:10.1167/tvst.15.5.26)
Supplement: Supplement 5 [file tvst-15-5-26_s005.docx]

**Supplementary Table S1. Repeatability of Retinal Curvature and Choroidal Thickness Measurements at Ring 1 and Ring 6**

| **Eye** | | **Test 1** | | **Test 2** | | **Test 3** | | **Test 4** | | **Mean±SD** | |
| --- | --- | --- | --- | --- | --- | --- | --- | --- | --- | --- | --- |
| **RC 1 (Center 1)** | | | | | | | | | | | |
| Eye1 | | 0.61 | | 0.6 | | 0.61 | | 0.61 | | 0.607 ± 0.005 | |
| Eye2 | | 0.41 | | 0.41 | | 0.41 | | 0.43 | | 0.415 ± 0.01 | |
| Eye3 | | 0.9 | | 0.76 | | 0.91 | | 0.88 | | 0.862 ± 0.069 | |
| Eye4 | | 0.29 | | 0.28 | | 0.29 | | 0.29 | | 0.288 ± 0.005 | |
| Eye5 | | 0.38 | | 0.36 | | 0.37 | | 0.38 | | 0.372 ± 0.01 | |
| Eye6 | | 0.75 | | 0.77 | | 0.74 | | 0.82 | | 0.77 ± 0.036 | |
| Eye7 | | 0.61 | | 0.61 | | 0.6 | | 0.59 | | 0.602 ± 0.01 | |
| Eye8 | | 0.47 | | 0.42 | | 0.42 | | 0.41 | | 0.43 ± 0.027 | |
| Eye9 | | 0.66 | | 0.65 | | 0.66 | | 0.67 | | 0.66 ± 0.008 | |
| Eye10 | | 0.3 | | 0.3 | | 0.3 | | 0.29 | | 0.298 ± 0.005 | |
| **RC 6 (Center 1)** | | | | | | | | | | | |
| Eye1 | | 2.44 | | 2.41 | | 2.41 | | 2.42 | | 2.42 ± 0.014 | |
| Eye2 | | 2.84 | | 2.78 | | 2.83 | | 2.81 | | 2.815 ± 0.026 | |
| Eye3 | | 2.32 | | 2.33 | | 2.31 | | 2.33 | | 2.323 ± 0.01 | |
| Eye4 | | 2.92 | | 2.92 | | 2.91 | | 2.92 | | 2.918 ± 0.005 | |
| Eye5 | | 2.69 | | 2.73 | | 2.68 | | 2.69 | | 2.698 ± 0.022 | |
| Eye6 | | 2.31 | | 2.36 | | 2.37 | | 2.35 | | 2.348 ± 0.026 | |
| Eye7 | | 2.54 | | 2.53 | | 2.54 | | 2.5 | | 2.528 ± 0.019 | |
| Eye8 | | 2.85 | | 2.89 | | 2.81 | | 2.86 | | 2.852 ± 0.033 | |
| Eye9 | | 2.48 | | 2.46 | | 2.49 | | 2.46 | | 2.472 ± 0.015 | |
| Eye10 | | 2.92 | | 2.9 | | 2.84 | | 2.91 | | 2.892 ± 0.036 | |
| **RC 1 (Center 2)** | | | | | | | | | | | |
| Eye1 | | 0.45 | | 0.46 | | 0.45 | | 0.43 | | 0.448 ± 0.013 | |
| Eye2 | | 0.72 | | 0.68 | | 0.71 | | 0.7 | | 0.702 ± 0.017 | |
| Eye3 | | 0.23 | | 0.22 | | 0.23 | | 0.23 | | 0.228 ± 0.005 | |
| Eye4 | | 0.84 | | 0.71 | | 0.81 | | 0.8 | | 0.79 ± 0.056 | |
| Eye5 | | 0.61 | | 0.6 | | 0.58 | | 0.62 | | 0.602 ± 0.017 | |
| Eye6 | | 0.6 | | 0.62 | | 0.64 | | 0.6 | | 0.615 ± 0.019 | |
| Eye7 | | 0.4 | | 0.42 | | 0.4 | | 0.4 | | 0.405 ± 0.01 | |
| Eye8 | | 0.56 | | 0.56 | | 0.61 | | 0.57 | | 0.575 ± 0.024 | |
| Eye9 | | 0.37 | | 0.34 | | 0.36 | | 0.36 | | 0.357 ± 0.013 | |
| Eye10 | | 0.21 | | 0.21 | | 0.2 | | 0.21 | | 0.208 ± 0.005 | |
| **RC 6 (Center 2)** | | | | | | | | | | | |
| Eye1 | | 2.91 | | 2.9 | | 2.94 | | 2.9 | | 2.912 ± 0.019 | |
| Eye2 | | 2.3 | | 2.29 | | 2.3 | | 2.3 | | 2.298 ± 0.005 | |
| Eye3 | | 1.97 | | 2.03 | | 2.01 | | 2.04 | | 2.013 ± 0.031 | |
| Eye4 | | 1.42 | | 1.42 | | 1.41 | | 1.4 | | 1.412 ± 0.01 | |
| Eye5 | | 2.73 | | 2.75 | | 2.73 | | 2.72 | | 2.733 ± 0.013 | |
| Eye6 | | 2.33 | | 2.33 | | 2.25 | | 2.32 | | 2.308 ± 0.039 | |
| Eye7 | | 2.2 | | 2.21 | | 2.21 | | 2.21 | | 2.208 ± 0.005 | |
| Eye8 | | 2.56 | | 2.52 | | 2.51 | | 2.52 | | 2.528 ± 0.022 | |
| Eye9 | | 2.96 | | 2.9 | | 2.81 | | 2.92 | | 2.898 ± 0.063 | |
| Eye10 | | 1.94 | | 1.91 | | 1.96 | | 1.95 | | 1.94 ± 0.022 | |
| **CT 1 (Center 1)** | | | | | | | | | | | |
| Eye 1 | | 274 | | 269 | | 278 | | 276 | | 274.3 ± 3.8 | |
| Eye 2 | | 153 | | 147 | | 142 | | 140 | | 145.5 ± 5.7 | |
| Eye 3 | | 165 | | 160 | | 162 | | 160 | | 161.8 ± 2.4 | |
| Eye 4 | | 159 | | 174 | | 171 | | 170 | | 168.5 ± 6.8 | |
| Eye 5 | | 189 | | 180 | | 196 | | 185 | | 187.5 ± 6.6 | |
| Eye 6 | | 213 | | 205 | | 214 | | 210 | | 210.5 ± 4.1 | |
| Eye 7 | | 134 | | 128 | | 136 | | 132 | | 132.5 ± 3.4 | |
| Eye 8 | | 142 | | 148 | | 145 | | 144 | | 144.8 ± 2.6 | |
| Eye 9 | | 196 | | 194 | | 195 | | 197 | | 195.5 ± 1.3 | |
| Eye 10 | | 169 | | 171 | | 169 | | 172 | | 170.3 ± 1.5 | |
| **CT 6 (Center 1)** | | | | | | | | | | | |
| Eye 1 | | 1097 | | 1110 | | 1116 | | 1082 | | 1101.3 ± 15.5 | |
| Eye 2 | | 554 | | 551 | | 521 | | 562 | | 547.0 ± 17.6 | |
| Eye 3 | | 697 | | 701 | | 719 | | 686 | | 700.8 ± 13.8 | |
| Eye 4 | | 581 | | 593 | | 601 | | 603 | | 594.5 ± 10.2 | |
| Eye 5 | | 681 | | 696 | | 658 | | 655 | | 672.5 ± 19.4 | |
| Eye 6 | | 519 | | 527 | | 518 | | 512 | | 519.0 ± 6.3 | |
| Eye 7 | | 431 | | 425 | | 448 | | 431 | | 433.8 ± 9.5 | |
| Eye 8 | | 616 | | 624 | | 633 | | 632 | | 626.3 ± 7.8 | |
| Eye 9 | | 850 | | 909 | | 909 | | 896 | | 891.0 ± 28.2 | |
| Eye 10 | | 851 | | 835 | | 840 | | 839 | | 841.3 ± 6.8 | |
| **CT 1 (Center 2)** | | | | | | | | | | | |
| Eye 1 | | 214 | | 220 | | 212 | | 218 | | 216.0 ± 3.6 | |
| Eye 2 | | 138 | | 133 | | 141 | | 137 | | 137.3 ± 3.3 | |
| Eye 3 | | 159 | | 162 | | 157 | | 160 | | 159.5 ± 2.1 | |
| Eye 4 | | 208 | | 215 | | 212 | | 210 | | 211.3 ± 3.0 | |
| Eye 5 | | 219 | | 214 | | 223 | | 221 | | 219.3 ± 3.9 | |
| Eye 6 | | 298 | | 304 | | 296 | | 301 | | 299.8 ± 3.5 | |
| Eye 7 | | 176 | | 181 | | 178 | | 179 | | 178.5 ± 2.1 | |
| Eye 8 | | 135 | | 139 | | 134 | | 136 | | 136.0 ± 2.2 | |
| Eye 9 | | 191 | | 198 | | 195 | | 194 | | 194.5 ± 2.9 | |
| Eye 10 | | 168 | | 173 | | 170 | | 172 | | 170.8 ± 2.2 | |
| **CT 6 (Center 2)** | | | | | | | | | | | |
| Eye 1 | | 638 | | 644 | | 631 | | 640 | | 638.3 ± 5.4 | |
| Eye 2 | | 504 | | 511 | | 498 | | 507 | | 505.0 ± 5.4 | |
| Eye 3 | | 589 | | 582 | | 595 | | 590 | | 589.0 ± 5.4 | |
| Eye 4 | | 582 | | 588 | | 575 | | 579 | | 581.0 ± 5.5 | |
| Eye 5 | | 925 | | 932 | | 914 | | 918 | | 922.3 ± 7.6 | |
| Eye 6 | | 1180 | | 1185 | | 1165 | | 1164 | | 1173.5 ± 10.6 | |
| Eye 7 | | 538 | | 533 | | 530 | | 542 | | 535.8 ± 5.2 | |
| Eye 8 | | 524 | | 533 | | 536 | | 517 | | 527.5 ± 8.5 | |
| Eye 9 | | 682 | | 694 | | 693 | | 692 | | 690.3 ± 5.7 | |
| Eye 10 | | 753 | | 754 | | 707 | | 732 | | 736.5 ± 20.8 | |

Repeatability of retinal curvature (RC) and choroidal thickness (CT) measurements was assessed in 10 eyes with repeated OCT scans. Measurements were obtained from the same retinal locations during three consecutive scans, with an additional scan performed on the following day. Results are shown for RC and CT at Ring 1 and Ring 6 across two scan centers. Mean ± standard deviation (SD) values are reported for each eye. Intraclass correlation coefficients (ICCs) indicated excellent repeatability across measurements (all ICCs > 0.98). One-way analysis of variance (ANOVA) showed no evidence of systematic differences across repeated measurements (F = 0.005–0.026; all P > 0.99).

**Abbreviations: RC** = Retinal Curvature; CT = choroidal thickness; OCT = optical coherence tomography; ICC = intraclass correlation coefficient; SD = standard deviation.
