# Supplementary material for: Associations of Retinal Curvature With Choroidal Thickness and OCTA-Derived Choroidal Flow-Density Metric in High Myopia: A Two-Center OCTA Study of Interocular Asymmetry
Source: Transl Vis Sci Technol. 2026 May 28;15(5):26. doi: 10.1167/tvst.15.5.26 (PMC13225303; doi:10.1167/tvst.15.5.26)
Supplement: Supplement 6 [file tvst-15-5-26_s006.docx]

**Supplementary Table S2. Interocular Associations Between Retinal Curvature Differences and Choroidal Metrics**

| **Ring** | **β (95% CI)** | ***P* value** | **q value (FDR)** |
| --- | --- | --- | --- |
| ΔCT(n=144) | | | |
| Ring 1 | -69.33 (-119.90, -18.76) | **0.008** | **0.01** |
| Ring 2 | -100.85 (-170.29, -31.41) | **0.005** | **0.01** |
| Ring 3 | -96.68 (-145.83, -47.53) | **<0.001** | **0.001** |
| Ring 4 | -62.90 (-113.50, -12.31) | **0.016** | **0.016** |
| Ring 5 | -65.30 (-112.15, -18.44) | **0.007** | **0.01** |
| Ring 6 | -58.44 (-91.59, -25.28) | **<0.001** | **0.002** |
| ΔCF(n=144) | | | |
| Ring 1 | 7.28 (-7.00, 21.57) | 0.319 | 0.525 |
| Ring 2 | 1.07 (-9.80, 11.95) | 0.847 | 0.847 |
| Ring 3 | 1.96 (-2.98, 6.90) | 0.438 | 0.525 |
| Ring 4 | 2.54 (-1.81, 6.90) | 0.255 | 0.525 |
| Ring 5 | -1.94 (-6.83, 2.94) | 0.437 | 0.525 |
| Ring 6 | -2.82 (-6.64, 1.01) | 0.151 | 0.525 |

Linear regression models were fitted using interocular differences (Δ = long eye − short eye) to evaluate within-subject associations between retinal curvature (ΔRC) and choroidal thickness (ΔCT) or choroidal flow-density metric (ΔCF). All models were adjusted for interocular axial length difference (ΔAL) and study center. Regression coefficients (β) represent the change in ΔCT (µm) or ΔCF per unit increase in ΔRC (10⁻² mm⁻²). P values were corrected for multiple comparisons across rings using the false discovery rate (FDR) method. Significant associations after FDR correction are indicated by q < 0.05.

Abbreviations: RC = retinal curvature; CT = choroidal thickness; CF = OCTA-derived choroidal flow-density metric; Δ = interocular difference (long eye − short eye); AL = axial length; CI = confidence interval; FDR = false discovery rate.
