# Supplementary material for: Associations of Retinal Curvature With Choroidal Thickness and OCTA-Derived Choroidal Flow-Density Metric in High Myopia: A Two-Center OCTA Study of Interocular Asymmetry
Source: Transl Vis Sci Technol. 2026 May 28;15(5):26. doi: 10.1167/tvst.15.5.26 (PMC13225303; doi:10.1167/tvst.15.5.26)
Supplement: Supplement 7 [file tvst-15-5-26_s007.docx]

**Supplementary Table S3. Quadrant-Based Exploratory Analyses of Retinal Curvature Associations With Choroidal Thickness and Choroidal Flow-Density Metric**

1. **Eye-level GEE results by quadrant**

| **Ring** | **β (95% CI)** | ***P* value** | **q value (FDR)** |
| --- | --- | --- | --- |
| RC main effect (β₁) | | | |
| Choroidal Thickness (CT) | | | |
| Ring 1 | −158.83 (−221.62, −96.04) | **<0.001** | **<0.001** |
| Ring 2 | −98.77 (−148.83, −48.71) | **<0.001** | **<0.001** |
| Ring 3 | −187.88 (−253.90, −121.86) | **<0.001** | **<0.001** |
| Ring 4 | −130.89 (−180.39, −81.39) | **<0.001** | **<0.001** |
| Ring 5 | −144.47 (−210.68, −78.27) | **<0.001** | **<0.001** |
| Ring 6 | −65.92 (−123.54, −8.31) | **0.025** | **0.025** |
| Choroidal Flow-Density Metric (CF) | | | |
| Ring 1 | 0.77 (−3.23, 4.77) | 0.705 | 0.705 |
| Ring 2 | −7.42 (−14.15, −0.70) | **0.031** | **0.046** |
| Ring 3 | 7.11 (2.45, 11.77) | **0.003** | **0.008** |
| Ring 4 | 7.66 (2.48, 12.85) | **0.004** | **0.008** |
| Ring 5 | 2.50 (−1.60, 6.16) | 0.181 | 0.217 |
| Ring 6 | 8.75 (3.63, 13.85) | **<0.001** | **0.005** |
| RC × HM interaction (β₃) | | | |
| Choroidal Thickness (CT) | | | |
| Ring 1 | 3.84 (−113.20, 120.87) | 0.949 | 0.949 |
| Ring 2 | −6.52 (−80.09, 67.05) | 0.862 | 0.949 |
| Ring 3 | −7.92 (−111.74, 95.90) | 0.881 | 0.949 |
| Ring 4 | 41.36 (−14.07, 96.80) | 0.144 | 0.463 |
| Ring 5 | 75.72 (−48.35, 199.79) | 0.232 | 0.463 |
| Ring 6 | −48.18 (−124.71, 28.35) | 0.463 | 0.463 |
| Choroidal Flow-Density Metric (CF) | | | |
| Ring 1 | −0.87 (−6.71, 4.97) | 0.771 | 0.935 |
| Ring 2 | −2.74 (−11.47, 5.99) | 0.539 | 0.935 |
| Ring 3 | −2.26 (−11.08, 6.55) | 0.615 | 0.935 |
| Ring 4 | −0.31 (−7.85, 7.22) | 0.935 | 0.935 |
| Ring 5 | −0.29 (−6.19, 5.62) | 0.924 | 0.935 |
| Ring 6 | −6.80 (−14.10, 0.49) | 0.068 | 0.406 |

1. **Interocular Δ results by quadrant**

| **Quadrant** | **ΔCT**  **β (95% CI)** | ***P***  **value** | **q value (FDR)** | **ΔCF**  **β (95% CI)** | ***P* value** | **q value (FDR)** |
| --- | --- | --- | --- | --- | --- | --- |
| I | −116.58  [−189.75, −43.41] | **0.002** | **0.004** | −2.91  [−8.39, 2.57] | 0.3 | 0.45 |
| N | −55.41  [−109.92, −0.89] | **0.048** | **0.048** | −3.64  [−9.70, 2.42] | 0.242 | 0.45 |
| S | −132.68  [−194.94, −70.43] | **<0.001** | **<0.001** | 9.18  [2.63, 15.73] | **0.007** | **0.021** |
| S I | −77.64  [−126.08, −29.19] | **0.002** | **0.004** | 12.08  [4.61, 19.55] | **0.002** | **0.011** |
| S II | −72.71  [−123.14, −22.29] | **0.005** | **0.008** | −0.77  [−4.92, 3.38] | 0.717 | 0.717 |
| T | −56.08  [−111.18, −0.99] | **0.048** | **0.048** | 4.11  [−5.53, 13.76] | 0.405 | 0.486 |

Exploratory quadrant-based analyses evaluating the associations between retinal curvature (RC) and choroidal metrics. Quadrants included nasal (N), temporal (T), superior (S), inferior (I), and two superoinferior sectors (S I and S II).**Eye-level analyses** (upper panels/tables) used generalized estimating equation (GEE) models with participant ID as the clustering variable (exchangeable working correlation) to account for inter-eye correlation. Models included RC (main effect) and the RC×high myopia (HM) interaction term, adjusted for age, sex, axial length, and study center.**Interocular difference (Δ) analyses** (lower panels/tables) were conducted at the participant level using Δ = (long eye − short eye), adjusted for interocular axial length difference (ΔAL) and study center.Results are reported as β (95% CI), two-sided P values, and false discovery rate (FDR)–adjusted q values. Given the exploratory nature and multiple comparisons, findings should be interpreted cautiously.

**Abbreviations: RC = retinal curvature; CT = choroidal thickness; CF =** OCTA-derived choroidal flow-density metric**; HM = high myopia; GEE = generalized estimating equation; Δ = interocular difference (long eye − short eye); ΔAL = interocular axial length difference; CI = confidence interval; FDR = false discovery rate; N = nasal; T = temporal; S = superior; I = inferior; S I/S II = superoinferior sectors.**
