# Supplementary material for: Associations of Retinal Curvature With Choroidal Thickness and OCTA-Derived Choroidal Flow-Density Metric in High Myopia: A Two-Center OCTA Study of Interocular Asymmetry
Source: Transl Vis Sci Technol. 2026 May 28;15(5):26. doi: 10.1167/tvst.15.5.26 (PMC13225303; doi:10.1167/tvst.15.5.26)
Supplement: Supplement 8 [file tvst-15-5-26_s008.docx]

**Supplementary Table S4. Summed Central, Peripheral, and Total Indices for Retinal Curvature Associations With Choroidal Metrics**

| **Outcome measure** | **Region** | **β (95% CI)** | ***P* value** | **q value (FDR)** |
| --- | --- | --- | --- | --- |
| **Eye-level analysis** | | | | |
| RC main effect (β₁) | | | | |
| CT index | Central | −148.96 [−202.93, −94.99] | <0.001 | <0.001 |
| CT index | Peripheral | −81.24 [−163.29, 0.80] | 0.052 | 0.052 |
| CT index | Total | −169.59 [−236.13, −103.05] | <0.001 | <0.001 |
| CF index | Central | 6.55 [2.70, 10.41] | <0.001 | 0.003 |
| CF index | Peripheral | −3.06 [−7.11, 0.98] | 0.138 | 0.138 |
| CF index | Total | 3.63 [−0.17, 7.44] | 0.061 | 0.092 |
| RC × High Myopia interaction (β₃) | | | | |
| CT index | Central | 55.27 [−78.61, 129.15] | 0.143 | 0.308 |
| CT index | Peripheral | −84.76 [−215.98, 46.47] | 0.206 | 0.308 |
| CT index | Total | −12.15 [−110.84, 86.54] | 0.809 | 0.809 |
| CF index | Central | −3.59 [−9.51, 2.34] | 0.235 | 0.353 |
| CF index | Peripheral | 0.39 [−5.95, 6.73] | 0.905 | 0.905 |
| CF index | Total | −6.16 [−12.27, −0.05] | 0.048 | 0.144 |
| **Interocular (Δ) analysis** | | | | |
| RC main effect (β₁) | | | | |
| ΔCT index | Central | −101.65 [−156.29, −47.01] | <0.001 | <0.001 |
| ΔCT index | Peripheral | −84.43 [−134.75, −34.11] | 0.001 | 0.001 |
| ΔCT index | Total | −128.21 [−187.30, −69.12] | <0.001 | <0.001 |
| ΔCF index | Central | 2.71 [−4.40, 9.81] | 0.456 | 0.528 |
| ΔCF index | Peripheral | −1.42 [−5.83, 2.99] | 0.528 | 0.528 |
| ΔCF index | Total | 2.12 [−4.01, 8.24] | 0.500 | 0.528 |

Analyses using central, peripheral, and total indices; N = 144 eyes (eye-level) or 144 paired eyes (Δ analysis).Analyses using summed indices derived from ring-wise measurements to provide a compact summary of central, peripheral, and total regions. For each metric, the central index was defined as the sum of Rings 1–3, the peripheral index as the sum of Rings 4–6, and the total index as the sum of Rings 1–6.Eye-level models were fitted using generalized estimating equations (GEE) with participant ID as the clustering variable (exchangeable working correlation), including the retinal curvature (RC) main effect and the RC×high myopia (HM) interaction term, adjusted for age, sex, axial length, and study center.**Interocular difference (Δ) models** were conducted at the participant level using Δ = (long eye − short eye), adjusted for interocular axial length difference (ΔAL) and study center.Results are reported as β (95% CI), two-sided P values, and false discovery rate (FDR)–adjusted q values. Because these indices are sums, they are presented primarily as sensitivity analyses to assess directional consistency with the ring-wise results.

Abbreviations: RC = retinal curvature; CT = choroidal thickness; CF = OCTA-derived choroidal flow-density metric; HM = high myopia; GEE = generalized estimating equation; Δ = interocular difference (long eye − short eye); AL = axial length; ΔAL = interocular axial length difference; CI = confidence interval; FDR = false discovery rate.
