# Supplementary material for: Associations of Retinal Curvature With Choroidal Thickness and OCTA-Derived Choroidal Flow-Density Metric in High Myopia: A Two-Center OCTA Study of Interocular Asymmetry
Source: Transl Vis Sci Technol. 2026 May 28;15(5):26. doi: 10.1167/tvst.15.5.26 (PMC13225303; doi:10.1167/tvst.15.5.26)
Supplement: Supplement 9 [file tvst-15-5-26_s009.docx]

****Supplementary Table S5. GEE-adjusted P values for differences between high myopia and non–high myopia****

| **Metric** | **Ring** | **Adjusted for age and sex** | **Adjusted for age, sex, and axial length** |
| --- | --- | --- | --- |
| RC | Ring 1 | 0.856 | 0.806 |
| RC | Ring 3 | 0.825 | 0.932 |
| RC | Ring 6 | <0.001 | 0.596 |
| CT | Ring 1 | <0.001 | 0.144 |
| CT | Ring 3 | <0.001 | 0.231 |
| CT | Ring 6 | <0.001 | 0.688 |
| CF | Ring 1 | 0.003 | 0.609 |
| CF | Ring 3 | 0.97 | 0.246 |
| CF | Ring 6 | 0.005 | 0.087 |

Generalized estimating equation (GEE)–based P values for differences in retinal curvature (RC), choroidal thickness (CT), and choroidal flow-density metric (CF), a device-derived OCTA metric between highly myopic and non–highly myopic eyes across macular rings.Models were adjusted for age and sex, with additional adjustment for axial length in sensitivity analyses (GEE+AL).

Abbreviations: RC = retinal curvature; CT = choroidal thickness; CF = OCTA-derived choroidal flow-density metric; GEE = generalized estimating equation; AL = axial length.
