# Supplementary material for: Associations of Retinal Curvature With Choroidal Thickness and OCTA-Derived Choroidal Flow-Density Metric in High Myopia: A Two-Center OCTA Study of Interocular Asymmetry
Source: Transl Vis Sci Technol. 2026 May 28;15(5):26. doi: 10.1167/tvst.15.5.26 (PMC13225303; doi:10.1167/tvst.15.5.26)
Supplement: Supplement 10 [file tvst-15-5-26_s010.docx]

### ****Supplementary Table S6. Spearman Correlations Between Interocular Differences in Retinal Curvature and Choroidal Thickness****

| **Ring** | **Total** | **Spearman ρ** | ***P* value** | **q value (FDR)** |
| --- | --- | --- | --- | --- |
| Ring 1 | 144 | -0.284 | **<0.001** | **<0.001** |
| Ring 3 | 144 | -0.331 | **<0.001** | **<0.001** |
| Ring 6 | 144 | 0.216 | **0.009** | **0.009** |

### Spearman rank correlations (ρ) between interocular differences in retinal curvature (ΔRC) and interocular differences in choroidal thickness (ΔCT) across selected concentric rings (Ring 1, Ring 3, and Ring 6). Interocular differences were computed as **Δ = (long eye − short eye)** at the participant level, where the long eye was defined as the eye with longer axial length. P values are two-sided. False discovery rate (FDR)–adjusted q values were calculated using the Benjamini–Hochberg procedure across the rings shown.

# Abbreviations: ΔRC = interocular difference in retinal curvature;ΔCT = interocular difference in choroidal thickness;AL = axial length;FDR = false discovery rate.
