# Supplementary material for: Associations of Retinal Curvature With Choroidal Thickness and OCTA-Derived Choroidal Flow-Density Metric in High Myopia: A Two-Center OCTA Study of Interocular Asymmetry
Source: Transl Vis Sci Technol. 2026 May 28;15(5):26. doi: 10.1167/tvst.15.5.26 (PMC13225303; doi:10.1167/tvst.15.5.26)
Supplement: Supplement 11 [file tvst-15-5-26_s011.docx]

## ****Supplementary Table S7. Sensitivity Analyses for Ring 6 Spearman Correlation Between ΔRC6 and ΔCT6****

| **Method** | **Total** | **Spearman ρ** | ***P* value** |
| --- | --- | --- | --- |
| Original | 144 | 0.216 | **0.009** |
| Trim 1% (two-sided) | 138 | 0.215 | **0.011** |
| Trim 2% (two-sided) | 133 | 0.207 | **0.017** |
| Winsor 1% (two-sided) | 144 | 0.216 | **0.009** |
| Winsor 2% (two-sided) | 144 | 0.216 | **0.009** |

Sensitivity analyses evaluating the robustness of the Spearman rank correlation between ΔRC6 and ΔCT6 after handling extreme observations. Results are shown for the original data and after trimming the most extreme 1%–2% of observations and after winsorizing the most extreme 1%–2%. P values are two-sided.

### Abbreviations: **ΔRC6** = interocular difference in retinal curvature at Ring 6; **ΔCT6** = interocular difference in choroidal thickness at Ring 6.
