# Supplementary material for: Associations of Retinal Curvature With Choroidal Thickness and OCTA-Derived Choroidal Flow-Density Metric in High Myopia: A Two-Center OCTA Study of Interocular Asymmetry
Source: Transl Vis Sci Technol. 2026 May 28;15(5):26. doi: 10.1167/tvst.15.5.26 (PMC13225303; doi:10.1167/tvst.15.5.26)
Supplement: Supplement 12 [file tvst-15-5-26_s012.docx]

**Supplementary Table S8. P Values for Retinal Curvature × Center Interaction Terms**

| **Outcome measure** | **Retinal Ring** | ***P*** for RC × Center Interaction |
| --- | --- | --- |
| CT | Ring 1 | 0.727 |
| CT | Ring 3 | 0.155 |
| CT | Ring 6 | 0.283 |
| CF | Ring 3 | 0.558 |
| CF | Ring 6 | 0.623 |

Interaction terms were derived from generalized estimating equation models adjusted for age, sex, axial length, and within-subject correlation between fellow eyes. No interaction terms reached statistical significance.

**Abbreviations:**RC = retinal curvature; CT = choroidal thickness; CF = OCTA-derived choroidal flow-density metric.
