# Supplementary material for: Associations of Retinal Curvature With Choroidal Thickness and OCTA-Derived Choroidal Flow-Density Metric in High Myopia: A Two-Center OCTA Study of Interocular Asymmetry
Source: Transl Vis Sci Technol. 2026 May 28;15(5):26. doi: 10.1167/tvst.15.5.26 (PMC13225303; doi:10.1167/tvst.15.5.26)
Supplement: Supplement 13 [file tvst-15-5-26_s013.docx]

**Supplementary Table S9. Standardized effect sizes of retinal curvature for choroidal thickness and choroidal flow-density metric**

| **Outcome measure** | **Ring** | **RC SD** | **RC IQR** | **Original**  **β (95% CI)** | **Effect per SD**  **β (95% CI)** | **Effect per IQR**  **β (95% CI)** | **P value** |
| --- | --- | --- | --- | --- | --- | --- | --- |
| CT | Ring 1 | 0.174 | 0.203 | −122.82 [−174.24, −71.40] | −21.3 [−30.3, −12.4] | −24.9 [−35.3, −14.5] | <0.001 |
| CT | Ring 3 | 0.578 | 0.66 | −136.62 [−186.76, −86.47] | −78.9 [−107.9, −49.9] | −90.2 [−123.3, −57.1] | <0.001 |
| CT | Ring 6 | 0.467 | 0.54 | 15.68 [−28.08, 59.43] | 7.3 [−13.1, 27.8] | 8.5 [−15.2, 32.1] | 0.482 |
| CF | Ring 3 | 0.578 | 0.66 | 5.56 [2.28, 8.83] | 3.2 [1.3, 5.1] | 3.7 [1.5, 5.8] | <0.001 |
| CF | Ring 6 | 0.467 | 0.54 | −5.07 [−8.33, −1.81] | −2.4 [−3.9, −0.8] | −2.7 [−4.5, −1.0] | 0.002 |

Values represent regression coefficients (β) with 95% confidence intervals derived from generalized estimating equation models. Effect sizes are expressed per one standard deviation (SD) and per interquartile range (IQR) increase in retinal curvature, using ring-specific distributions estimated from the study sample. Models were adjusted for age, sex, axial length, and imaging center. P values were adjusted for multiple comparisons using the Benjamini–Hochberg false discovery rate procedure.

**Abbreviations:** RC = retinal curvature;CT = choroidal thickness;CF = OCTA-derived choroidal flow-density metric;SD = standard deviation;IQR = interquartile range;CI = confidence interval;FDR = false discovery rate.
