# Supplementary material for: Associations of Retinal Curvature With Choroidal Thickness and OCTA-Derived Choroidal Flow-Density Metric in High Myopia: A Two-Center OCTA Study of Interocular Asymmetry
Source: Transl Vis Sci Technol. 2026 May 28;15(5):26. doi: 10.1167/tvst.15.5.26 (PMC13225303; doi:10.1167/tvst.15.5.26)
Supplement: Supplement 14 [file tvst-15-5-26_s014.docx]

**Supplementary Table S10. Sensitivity analyses of the RC–choroidal associations after substituting axial length with spherical equivalent**

| **Model term** | **Ring 1**  **β (95% CI)** | ***P* value** | **q value (FDR)** | **Ring 3**  **β (95% CI)** | ***P* value** | **q value (FDR)** | **Ring 6**  **β (95% CI)** | ***P* value** | **q value (FDR)** |
| --- | --- | --- | --- | --- | --- | --- | --- | --- | --- |
| **CT(Model 1)** | | | | | | | | | |
| RC main effect (β₁) | −122.82 [−174.24, −71.40] | <0.001 | <0.001 | −136.62  [−186.76, −86.47] | <0.001 | <0.001 | 15.68  [−28.08, 59.43] | 0.482 | 0.482 |
| RC × HM interaction (β₃) | 61.45  [−12.58, 135.49] | 0.104 | 0.156 | 30.14  [−34.94, 95.21] | 0.364 | 0.364 | −105.83  [−201.77, −9.89] | 0.031 | 0.092 |
| **CF(Model 1)** | | | | | | | | | |
| RC main effect (β₁) | 7.24  [−2.19, 16.68] | 0.132 | 0.132 | 5.56  [2.28, 8.83] | <0.001 | 0.003 | −5.07  [−8.33, −1.81] | 0.002 | 0.003 |
| RC × HM interaction (β₃) | −1.28  [−14.05, 11.49] | 0.844 | 0.844 | −6.12  [−11.35, −0.89] | 0.022 | 0.065 | 6.81  [−0.39, 14.02] | 0.064 | 0.096 |
| **CT(Model 2)** | | | | | | | | | |
| RC main effect (β₁) | −130.24 [−189.95, −70.52] | <0.001 | <0.001 | −146.96  [−204.81, −89.11] | <0.001 | <0.001 | 39.56  [−4.09, 83.20] | 0.076 | 0.076 |
| RC × HM interaction (β₃) | 81.26  [−1.93, 164.45] | 0.056 | 0.096 | 48.14  [−24.57, 120.84] | 0.194 | 0.194 | −105.33  [−216.73, 6.07] | 0.064 | 0.096 |
| **CF(Model 2)** | | | | | | | | | |
| RC main effect (β₁) | 7.08  [−2.57, 16.74] | 0.15 | 0.15 | 5.76 [2.31, 9.22] | 0.001 | 0.003 | −4.81  [−8.04, −1.59] | 0.003 | 0.005 |
| RC × HM interaction (β₃) | −2.36  [−15.39, 10.68] | 0.723 | 0.723 | −6.52  [−11.86, −1.18] | 0.017 | 0.05 | 6.74  [−0.45, 13.93] | 0.066 | 0.099 |
| **CT(Model 3)** | | | | | | | | | |
| RC main effect (β₁) | −123.04  [−174.37, −71.71] | <0.001 | <0.001 | −137.53  [−187.25, −87.82] | <0.001 | <0.001 | 13.81  [−30.14, 57.76] | 0.538 | 0.538 |
| RC × HM interaction (β₃) | 60.55  [−14.68, 135.79] | 0.115 | 0.172 | 29.16  [−36.61, 94.94] | 0.385 | 0.385 | −104.49  [−200.77, −8.22] | 0.033 | 0.1 |
| **CF(Model 3)** | | | | | | | | | |
| RC main effect (β₁) | 7.32  [−2.22, 16.85] | 0.133 | 0.133 | 5.76  [2.48, 9.04] | <0.001 | 0.002 | −4.99  [−8.37, −1.61] | 0.004 | 0.006 |
| RC × HM interaction (β₃) | −1.20  [−14.04, 11.64] | 0.854 | 0.854 | −6.04  [−11.25, −0.83] | 0.023 | 0.069 | 6.75  [−0.49, 14.00] | 0.068 | 0.102 |

Eye-level generalized estimating equation (GEE) models with exchangeable working correlation were used, accounting for within-subject correlation (two eyes per participant; clustered by participant ID). Models were adjusted for age, sex, and study center. Sensitivity analyses evaluated robustness of the retinal curvature (RC) association with choroidal thickness (CT) and choroidal flow-density metric (CF) by substituting axial length (AL) with spherical equivalent (SE) and/or including both refractive metrics in the model (as specified below). Results are presented for Rings 1, 3, and 6 as β coefficients with 95% confidence intervals. P values are shown with false discovery rate (FDR)–adjusted q values within each outcome across rings.

# Abbreviations: **AL** = axial length; **CF** = OCTA-derived choroidal flow-density metric; **CI** = confidence interval; **CT** = choroidal thickness; **FDR** = false discovery rate; **GEE** = generalized estimating equation; **HM** = high myopia; **RC** = retinal curvature; **SE** = spherical equivalent.
